# COVID-19 Factors and Psychological Factors Associated with Elevated Psychological Distress among Dentists and Dental Hygienists in Israel

**DOI:** 10.3390/ijerph17082900

**Published:** 2020-04-22

**Authors:** Maayan Shacham, Yaira Hamama-Raz, Roni Kolerman, Ori Mijiritsky, Menachem Ben-Ezra, Eitan Mijiritsky

**Affiliations:** 1 School of Social Work, Ariel University, Ariel 40700, Israel; drmaayanshacham@gmail.com (M.S.); razizik@bezeqint.net (Y.H.-R.); menbe@ariel.ac.il (M.B.-E.); 2 Department of Periodontology and Dental Implantology, The Maurice and Gabriela Goldschleger School of Dental Medicine, Tel Aviv University, Tel Aviv 6997801, Israel; Kolerman@netvision.net.il; 3 Department of Psychology, Tel Aviv-Yafo Academic College, Tel Aviv 6818543, Israel; orimijiritsky2@gmail.com; 4 Department of Otolaryngology, Head and Neck and Maxillofacial Surgery, Tel-Aviv Sourasky Medical Center, Sackler Faculty of Medicine, Tel Aviv 6139001, Israel; 5 The Maurice and Gabriela Goldschleger School of Dental Medicine, Tel Aviv University, Tel Aviv 6997801, Israel

**Keywords:** COVID-19, dentists, dental hygienists, psychological factors, psychological distress

## Abstract

The aim of this study was to evaluate the association of COVID-19 factors and psychological factors with psychological distress among dental staff during the COVID-19 pandemic outbreak. A cross-sectional survey was conducted among 338 Israeli dentists and dental hygienists, who provided their demographic data; answered questions about COVID-19-related factors; and were assessed by subjective overload, self-efficacy, and psychological distress scales. Data were analyzed using a multivariate logistic regression. Results revealed that elevated psychological distress was found among those who have background illness, fear of contracting COVID-19 from patient, and a higher subjective overload. Lower psychological distress was associated with being in a committed relationship and having higher scores for self-efficacy. Given these results, gathered during times of an infectious disease outbreak, exploring psychological distress among dental staff is warranted as the effects may be long-term.

## 1. Introduction

In December 2019, the coronavirus (COVID-19) outbreak emerged in Wuhan City, China [1]. On 30 January, 2020, the World Health Organization (WHO) declared COVID-19 to be a pandemic.

Dental staff, like other healthcare providers, may be exposed to COVID-19 as part of their work, as the virus can spread from person to person through small droplets from the nose or mouth [2]. In dental practice, the possible routes of transmission for COVID-19, or SARS-CoV-2, include airborne spread via aerosols formed during dental procedures [3], contact spread, and contaminated surfaces spread [4]. Moreover, dental staff may be conflicted about performing their professional roles as health care providers versus their roles as family members (e.g., spouse/parents). According to Maunder et al. [5], the conflict of altruism and professional liability, on the one hand, with fear and blame for potentially endangering their relatives to a highly infectious agent, on the other hand, was a tremendous burden for many medical staff individuals during the severe acute respiratory syndrome (SARS) outbreak. 

In light of these concerns, psychological distress may be a possible psychological reaction that might cause a potential reduction in the quality of the treatment provided [6,7].

Previous studies, which were conducted when SARS emerged from Guangdong, China, in November 2002 and spread rapidly, revealed that significant emotional distress was present in 18%–57% of healthcare workers [8,9,10,11] and was associated with fear of contagion [12], concern for family [10,13], job stress [11], and attachment insecurity [14]. In addition, despite usage of protective measures, medical staff were infected with SARS during aerosol-producing procedures [14]. Approximately 30% of SARS-infected individuals were among medical staff, and in Canada alone, about 50% of the 182 SARS-infected individuals were medical staff, three of whom eventually died [15].

Given the above, and in line with the Israeli Ministry of Health orders, given on 17 March, 2020, to dental clinics in Israel to stop providing elective dental care and to provide treatments for dental emergencies only, this study aimed to explore psychological distress among dentists and dental hygienists in Israel during the COVID-19 pandemic outbreak. In addition, we were interested to explore several COVID-19-related factors and psychological factors which may be associated with psychological distress. 

The proposed factors stem from the transactional cognitive theory of stress and coping [16]. According to this theory, a state of duress is a dynamic state of imbalance between oneself and one’s surroundings, when the latter are perceived as placing too many demands on one’s personal well-being. Specifically, we explored COVID-19-related factors, namely being in a risk group, fear of contracting COVID-19 from patients, and receiving enough professional knowledge regarding COVID-19, and psychological factors, namely subjective overload and self-efficacy. 

Regarding the psychological factors, subjective overload relates to staff perceptions of their circumstances, which together with their coping strategies determine the level of their job stress. In reality, different staff members will experience different levels of stress in response to the same set of circumstances [17].

Self-efficacy relates to a person’s belief in his or her capability to perform at different levels in response to real-time events and to execute the different behaviors needed to achieve specific goals [18]. Individuals with strong self-efficacy select more challenging goals and tend to focus on opportunities, not on obstacles [19]. Studies highlight the importance of self-efficacy in the personal and professional development of an individual, as well as in their handling of stressful events and in overcoming traumas [20]. 

In summary, to the best of our knowledge, there are no studies regarding the association of COVID-19 factors and psychological factors with psychological distress among dental staff during the COVID-19 pandemic outbreak. This study sought to address these questions. 

## 2. Materials and Methods 

### 2.1. Sampling and Procedure

We used an internet platform to conduct the survey (www.imkforms.com, IMK, Rishon Lezion, Israel) after gaining approval for the study from the Institutional Review Board of the authors’ university. The participants were approached using social media, dedicated mailing lists, and forums. During the period of 30 March to 10 April, 2020, we collected data from dentists and dental hygienists in Israel (*n* = 338). The mean age of these participants was 46.39 years (SD = 11.18, range = 24–74), 58.6% were female (*n* = 198), 80.2% (*n* = 271) were in a committed relationship, and 58.6% were dentists (*n* = 198). Each participant signed an electronic informed consent form. 

### 2.2. Measurements

Beyond basic demographic details, the following self-reported measures were used: 

#### 2.2.1. COVID-19-Related Factors

Being in a risk group was measured by the question: “Are you defined as being in a high-risk population (suffering from chronic lung disease or moderate to severe asthma/chronic kidney disease and who are undergoing dialysis/liver disease/serious heart conditions/conditions that can cause a person to be immunocompromised, including cancer treatment/diabetes)?” The responses were coded as 0 for “No” and 1 for “Yes”, and 19.5% (*n* = 66) answered “Yes”.

Fear of contracting COVID-19 from patients was measured by the question: “Are you afraid to be infected with COVID-19 because of your profession?” Responses were coded from 0 for “Not at all” to 4 for “Very afraid”. The mean score of this item was 2.88 (SD = 0.88).

Receiving enough professional knowledge regarding COVID-19 was measured by the question: “Do you feel that you have acquired sufficient knowledge (lectures, seminars, information leaflet, etc.) regarding maintaining a safe working environment since the COVID-19 outbreak?” Responses were coded from 0 for “Not at all” to 4 for “Very much”. The mean score of this item was 2.66 (SD = 1.01).

#### 2.2.2. Psychological Factors 

Subjective overload was measured by the Demands Scale—Short Version [21]. These six items looking at various aspects of personnel stress are as follows: “1. I cannot handle the contradicting demands made on me during my work; 2. The amount of work time available to me is insufficient; 3. My job poses demands without having the right equipment and resources; 4. I never leave my work feeling that I have finished all my chores; 5. I am unable to perform my job to the best of my ability in the time allocated; 6. I am required to perform simple chores that prevent me from performing more sophisticated ones”. The responses were coded from 1 for “Not at all” to 5 for “Very much”. Cronbach’s Alpha for the demands scale in the current study was 0.87. 

Self-efficacy was measured by the General Self-Efficacy Scale [22]. This is a 10-item questionnaire answered on a four-category Likert scale ranging from 1 for “Strongly disagree” to 4 for “Strongly agree”. An example item is “I can always manage to solve difficult problems if I try hard enough”. Cronbach’s Alpha for the demands scale in the current study was 0.86. 

#### 2.2.3. Outcome Variable

Psychological distress was measured by Kessler’s K6 [23], which included items on feeling nervous, hopeless, restless/fidgety, depressed, like everything was an effort, and worthless during the previous 30 days. Scores ranged from 0 to 30, with 19 or higher indicating elevated psychological distress. Cronbach’s Alpha for the demands scale in the current study was 0.86. 

### 2.3. Statistical Analyses

Data were analyzed using a multivariate logistic regression [24] to measure the association of elevated psychological distress (K6 ≥ 19), as the outcome, with the following variables entering the equation: (1) Demographics (age, sex, relationship, profession); (2) COVID-19-related factors (being in a risk group, fear of contracting COVID-19 from patients, receiving enough professional knowledge regarding COVID-19); (3) Psychological factors (subjective overload, self-efficacy). For each variable, we calculated the odds ratio (OR) and 95% CI using SPSS version 25 (IBM, Armonk, NY, USA). 

## 3. Results

Risk of elevated psychological distress was found in 11.5% of the sample (*n* = 39). Elevated psychological distress was found among those who have background illness (OR = 3.023 (95% CI: 1.186–7.705); *p* = 0.021), fear of contracting COVID-19 from a patient (OR = 2.110 (95% CI: 1.236–3.603); *p* = 0.006), and higher subjective overload (OR = 1.073 (95% CI: 1.010–1.141); *p* = 0.022). However, lower psychological distress was associated with being in a committed relationship (OR = 3.023 (95% CI: 1.186–7.705); *p* = 0.021) and having higher self-efficacy (OR = 0.898 (95% CI: 0.833–0.968); *p* = 0.005). Table 1 contains further information. 

## 4. Discussion

This study provided an evaluation of the level of psychological distress experienced by Israeli dentists and dental hygienists during the COVID-19 pandemic outbreak and assessed possible factors that might be associated with it. The findings confirmed our hypothesis suggesting that dental staff would exhibit an elevated risk for developing psychological distress. Nevertheless, as the evaluation of psychological distress in the current study has taken place during the spread of COVID-19 in Israel, we cannot rule out the possibility for higher psychological distress rates in the long term, as was found previously among healthcare workers after the 2003 outbreak of SARS in Canada [25]. 

Furthermore, regarding the association of COVID-19 factors and psychological factors with elevated psychological distress, the findings indicate that among the COVID-19 factors, only the fear of contracting COVID-19 from a patient associated positively with elevated psychological distress. This may be supported by scientific evidence showing that there is an unwillingness on the part of dentists to treat patients with infectious diseases such as HIV [26,27] and tuberculosis [28]. With reference to the psychological factors, high subjective overload and a low score for self-efficacy were found to be associated with elevated psychological distress. The association of subjective overload with psychological distress might be explained through the Karasek’s job demand–control–support model [29] which argues that the experience of stress (stress outcomes) is a consequence of the interaction between the stressor (e.g., job demands), the individual’s perception of their control over the stressor (e.g., job control), and social support. As the on-going management of COVID-19 is full of uncertainty and characterized by the individual having low levels of control (along with social isolation, which prevent teamwork), it might be that dental staff are experiencing a higher than usual subjective overload.

Concerning self-efficacy, our results show that dental staff who exhibited a higher score for self-efficacy revealed lower psychological distress. Indeed, in stressful events, self-efficacy plays a crucial role, as at high levels it can enhance personal performance in different occupational tasks and related behaviors [30]. In accordance with this finding, self-efficacy among rescue workers was found to mediate the association between stress appraisal and compassion satisfaction [31]. Furthermore, general self-efficacy was found as a mediator for the association of daily life stressors with psychopathological symptoms and subjective well-being [32]. One possible explanation may stem from the Conservation of Resources (COR) theory [33,34] which proposes that individuals seek to build up and retain resources as a result of actual or threatened loss thereof, after coping with stressful circumstances. Resource gains can help to offset the impact of resource losses. As such, it might be that those with higher self-efficacy succeed in acquiring more resources to handle such situations and their consequences in the future. 

Finally, regarding the demographic data, our findings showed that dental staff participants who have background illnesses exhibited elevated psychological distress. This may be explained as individuals with such chronic illnesses might be more vulnerable if infected by COVID-19. According to data by the Centers for Disease Control and Prevention, people who have chronic medical conditions, such as diabetes, lung disease, and heart disease, face an increased chance of being hospitalized with COVID-19 and put into intensive care [35]. Thus, it is not surprising to find elevated psychological distress in those participants who cope with chronic illnesses. 

Another demographic datum that was associated with lower psychological distress was being in a committed relationship. This finding is supported by [36], which reports that as part of the mental health care for medical staff during the COVID-19 epidemic, the Chinese medical staff members were kept in contact with their families by video, in order to raise their morale. A possible explanation may be the buffering effect that family support provides in disruptive events, which is well documented in the scientific literature [37,38].

Limitations to this study include its cross-sectional design, which precludes causal inferences, and relatively low response rate, which may be explained by the short time period of study sampling. Nevertheless, the sample size may be considered to be moderate. In addition, the study is subject to selection bias and sampling error, as participants were approached using social media, dedicated mailing lists, and forums.

This study highlights the importance of considering the mental health of dental staff during times of infectious disease outbreak. Psychological distress among dental staff members may have long-term effects and, as such, will have future implications for training, such as the need for provision of mental health workshops for enhancing self-efficacy along with broadening mental health education as part of the core dental curriculum.

## 5. Conclusions

Overall, this study sheds light on the association of COVID-19 factors and psychological factors with elevated psychological distress among dental staff. Our results show that elevated psychological distress was found among those who have background illness, those who feared contracting COVID-19 from a patient, and those who had higher subjective overload. Lower psychological distress was associated with being in a committed relationship and having higher self-efficacy. These results highlight the importance of exploring psychological distress among dental staff as it may have long-term implications for personal and professional welfare. The introduction of methods for enhancing the self-efficacy of dental staff, along with providing a broader understanding of mental health, are recommended. Further research should examine the short-term and long-term psychological effects of the COVID-19 pandemic among dental staff.

## Figures and Tables

**Table 1:** Factors associated with psychological distress among dentists and dental hygienists.

	Sample (*n* = 338)	
Demographics	Adjusted OR (95% CI)	*p* Value
Age, Years	0.964 (0.929–1.001)	0.054
Sex, Female	1.331 (0.467–3.797)	0.593
In committed relationship, Yes	0.345 (0.147–0.810) *	0.014
Suffer from background illness, Yes	3.023 (1.186–7.705) *	0.021
**Dental Group**		
Dentist, Yes	0.905 (0.642–1.277)	0.570
**COVID-19-Related Aspects**		
Contracting COVID-19 from patient	2.110 (1.236–3.603) **	0.006
Received information about COVID-19	0.778 (0.529–1.144)	0.202
**Psychological Factors**		
Subjective overload	1.073 (1.010–1.141) *	0.022
Self-Efficacy	0.898 (0.833–0.968) **	0.005

* *p* ≤ 0.05; ** *p* ≤ 0.01.
